# Endoscopic retrieval of a proximally migrated pancreatic stent complicated by a roomy ampulla of Vater and duodenal diverticula

**DOI:** 10.1055/a-2512-4386

**Published:** 2025-02-11

**Authors:** Xu Ji, Can Sun, Zheng Zhang, Feng Du, Shutian Zhang, Peng Li

**Affiliations:** 1 26455 Department of Gastroenterology, Capital Medical University Affiliated Beijing Friendship Hospital, Beijing, China

Proximal migration of a pancreatic stent is a rare complication of endoscopic retrograde cholangiopancreatography (ERCP), with an incidence of approximately 5.2% 1 . This constitutes a dire scenario, as it may cause damage to the pancreatic duct, potentially necessitating surgical intervention. We describe a case of successful retrieval of a proximally migrated pancreatic stent using a retrieval basket in a roomy ampulla of Vater with duodenal diverticula.

A 72-year-old man underwent a routine ERCP for choledocholithiasis and had a 5-Fr single-pigtail pancreatic stent placed owing to difficult biliary cannulation. After 3 weeks, the pancreatic stent was not visible at the opening of the duodenal papilla; a plain radiograph showed that the stent had migrated into the pancreatic duct ( Fig. 1 ).

**Fig. 1:**
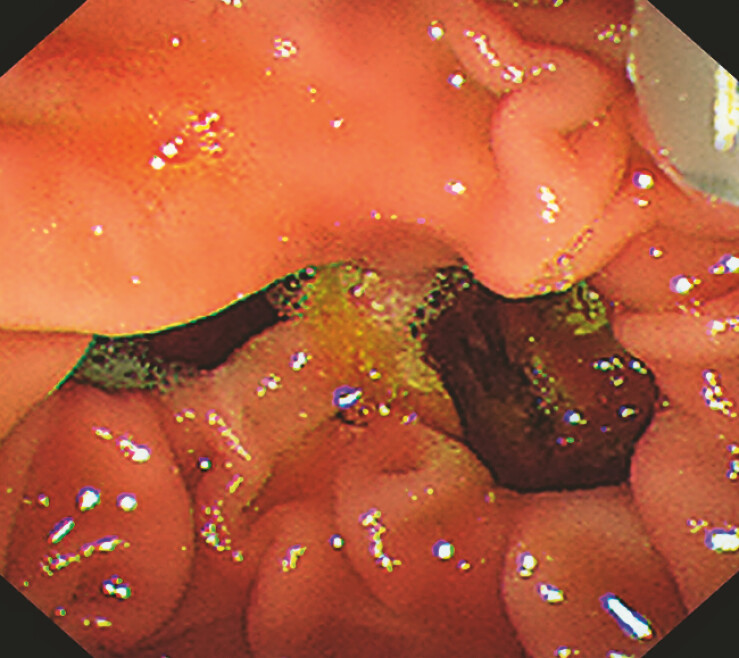
Endoscopic view of the duodenal papilla with juxtapapillary duodenal diverticula.

ERCP was performed with the aim of retrieving the migrated pancreatic stent ( Video 1 ). We were initially unable to grasp the stent using foreign body forceps (SSR-5; Cook Medical, Bloomington, Indiana, USA) because the ampulla of Vater was roomy, and 2D radiographic images were unable to play an influential guiding role ( Fig. 2 ). A subsequent attempt with an extraction balloon (Cook Medical) was unsuccessful, and the stent became folded ( Fig. 3 ). After this, pancreatoscopy was performed to ensure there was no visible damage to the duct ( Fig. 4 ). Finally, we used a retrieval basket (Olympus, Tokyo, Japan) to secure the stent and retrieve it from the pancreatic duct ( Fig. 5 ). The procedure was successful, and the patient was discharged without complications.

Endoscopic retrieval of a proximally migrated pancreatic stent in a roomy ampulla of Vater with duodenal diverticula.Video 1

**Fig. 2:**
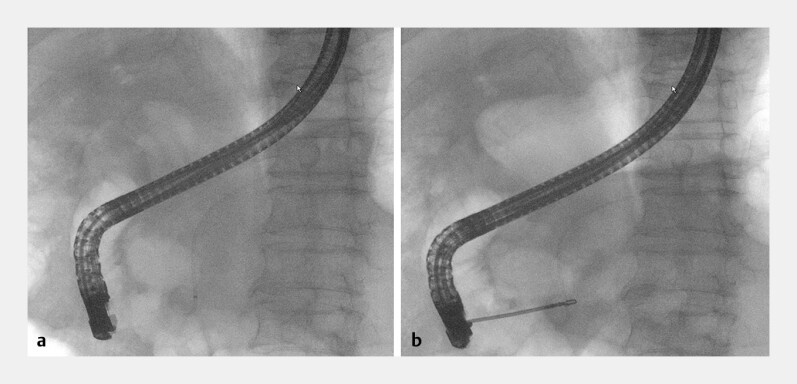
Fluoroscopic images showing: **a** complete migration of the pancreatic stent into the pancreatic duct; **b** foreign body forceps that are able to reach the distal end of the stent but not able to grasp it.

**Fig. 3:**
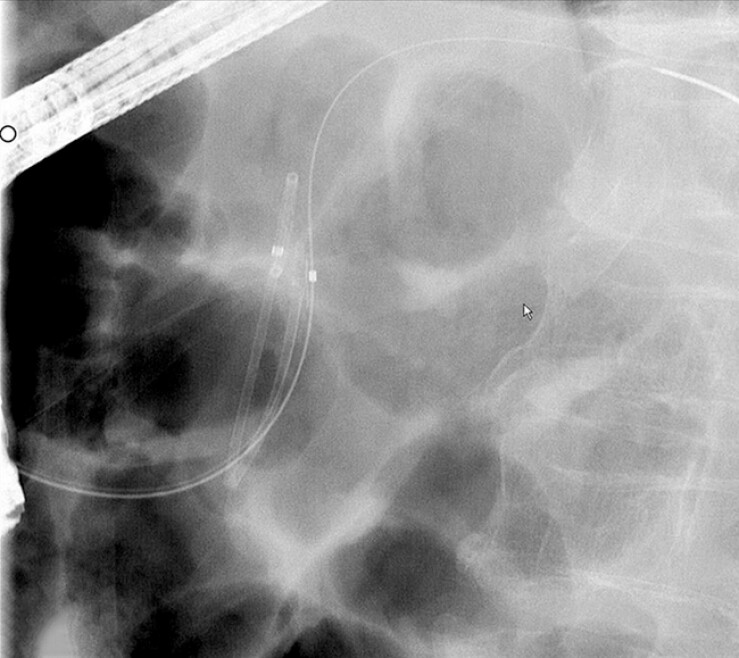
Fluoroscopic image showing the folded stent.

**Fig. 4:**
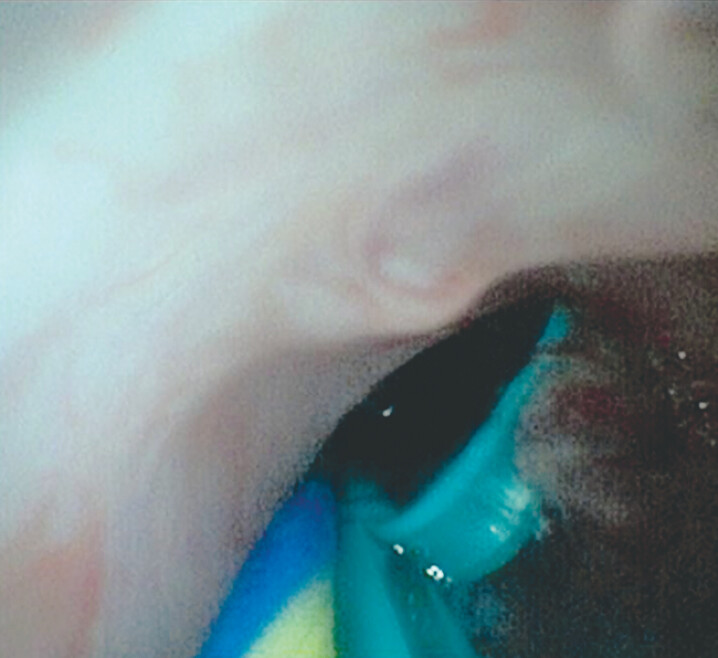
Choledochoscopic view showing no evidence of injury to the pancreatic duct.

**Fig. 5:**
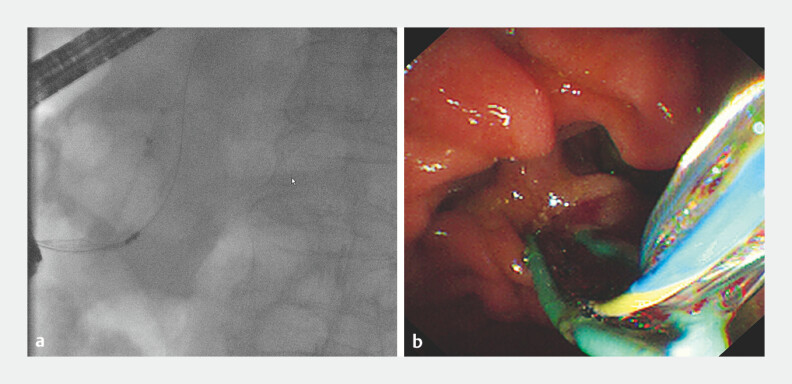
Images showing retrieval of the stent using the basket on: **a** fluoroscopic view; **b** endoscopic view.

The significant challenges in the present case were the roomy ampulla of Vater, in which the pigtail of the stent was located, and the narrow duodenal papilla with juxtapapillary duodenal diverticula. Foreign body forceps could not grasp the stent, as was reported in another case where a single-pigtail pancreatic stent was inserted in a normal-sized pancreatic duct 2 ; however, it was possible to use a basket to successfully grasp the pancreatic stent in the roomy ampulla of Vater.

Endoscopy_UCTN_Code_CPL_1AK_2AD

## Notes

Endoscopy E-Videos https://eref.thieme.de/e-videos
*E-Videos* is an open access online section of the journal *Endoscopy* , reporting on interesting cases and new techniques in gastroenterological endoscopy. All papers include a high-quality video and are published with a Creative Commons CC-BY license. Endoscopy E-Videos qualify for HINARI discounts and waivers and eligibility is automatically checked during the submission process. We grant 100% waivers to articles whose corresponding authors are based in Group A countries and 50% waivers to those who are based in Group B countries as classified by Research4Life (see: https://www.research4life.org/access/eligibility/ ). This section has its own submission website at https://mc.manuscriptcentral.com/e-videos .
